# Immunity in Tuberculosis

**Published:** 1900-11

**Authors:** A. D. Lake

**Affiliations:** Gowanda, N. Y.


### IMMUNITY IN TUBERCULOSIS.

By A. D. LAKE, M. D., Gowanda, N. Y.

IN THE rapid progress in medicine which the past decade has witnessed, only a few things have positively been made known to us regarding tuberculosis, first and foremost standing the unrivaled discovery of Koch—the bacillus. The growing, perhaps generally admitted, belief in the contagiousness of the malady is not new, as the same opinion was repeatedly expressed by observers many years since.

As to the therapeutics, who can say that today, except in a general way, guided by our improved knowledge of hygiene, foods, etc., and climatic conditions, remedial measures, as applied to the treatment of tuberculosis, are any more effective than those used by our forefathers. We still have no specific.

The great field which this disease in its various forms and manifestations opens up for study and investigation, although continually explored by the most earnest and skilful workers through all the past, has still reserved its great secrets, the revelation of which is to reward the labor of the students of the present, or of those who are to come after. In any attempt to discuss this subject, one is confronted at once with the impossibility of grasping more than a small portion of the matter. At the present time a few points in etiology and treatment are perhaps occupying more attention than others. No more important question demands our attention than the renewal of investigations as to the contagiousness of this malady. Admitting that sufficient evidence exists to warrant placing it among affections of this class, then we are given a new basis for study, inquiring first as to what other features it may have in common with most other infectious diseases. This question has been to the writer an interesting subject of investigation, and the possession of a comparatively large field for observation has led him to believe that tuberculosis is not only infectious, but that it is in other respects most analagous to this class of diseases.

The scope of this paper will not permit even touching upon all points of this similarity, but I desire briefly to submit to you my reasons for believing that tuberculosis, like several other infectious diseases, confers immunity; that occurring in glands, bones and joints, and possibly in mucous membrane, and being recovered from, affords protection from subsequent tuberculosis of the lungs. That this disease existing in mild form in parts of the body other than vital organs is susceptible of recovery, and with recovery produces immunity.

You will pardon me if I digress here for the purpose of giving a description of the locality where, and the people among whom my observations regarding this disease have been largely made. The Cattaraugus Reservation is situated in the valley of the Cattaraugus creek, extending up this valley from Lake Erie, twelve miles, and in width one and one-half miles each side of the creek, partly in Cattaraugus county and partly in Erie county, the stream being the boundary between the two counties. A part of the reservation is level, viz.: the flat lands on each side of the creek. On the south side of the creek and three-fourths of a mile distant, an elevation commences, which attains a height of 600 feet in four miles. The elevation is less on the north, but the Reservation is several hundred feet lower than any of the surrounding country. The creek contains a far less volume of water than formerly, and in its recession has left on each side a wide strip of flat land, the soil of which is alluvial and produces most abundant crops. This ancient river bottom is about 100 feet lower than the rest of the Reservation and is very thickly settled, the richness of the soil and the ease with which it is cultivated making it the favorite land of the Indian, while large tracts of land more elevated are entirely uninhabited. Probably three-fourths of the population live on the river bottom. The whole surface of the Reservation has been for many years nearly denuded of timber, nothing being left but small underbrush. The trees have been cut and sold for lumber. A large tract of beautiful pine has been disposed of in this manner.

The first point to attract the attention of one having occasion to look after the health of these people is the imperfect and poorly developed physique of the children, and also of the next preceding generation. This is especially characteristic of the males. The Indian women present a far more vigorous appearance and evidence of larger vitality; but the last two generations have not the endurance of their predecessors in either sex. A further study into their physical condition confirms this impression. On watching them engaged in a game of ball, it is seen that the violent exercise necessarily undergone is very fatiguing to them, and that after running rapidly for a few minutes, their respiration is increased to such an extent that it becomes fairly laborious. They frequently throw themselves on the ground, completely exhausted. The same is true in the dances that form a part of the pagan ceremonies, which are still observed to some extent among them. The surrounding farmers find it impossible to obtain the same amount of work from the Indians whom they employ that they get from white laborers, and this applies to the more industrious class who are willing to work. Instead of the full-chested, robust figure presented by the uncivilised Indian of a few generations past, we see the emaciated, stooping, and in all respects inferior, and degenerate successor of today.

Now, when is added to this condition of physical depravity, the fact of their miserable habitations, unhealthful sanitary surroundings, and their intemperance, we have all the factors needed for the propagation and increase of those diseases so prevalent among them. They pay little or no attention to personal cleanliness. They live largely upon a poorly cooked vegetable diet, the only meat with far the larger number being fat salt pork. Their houses are built slightly elevated from the ground, in many instances the cold air having free access beneath the floor. The rooms are small and ceilings low. A family of half a dozen are frequently crowded into a room twelve feet square, occupying it day and night.

A very interesting study presents itself to the philanthropist as well as to the physician, regarding the association of these causes with the present condition of the race, and the probability of improvement by their removal, if such removal be possible. Certainly with their continuation there can be no hope, and he who will be most useful in elevating the Indian to a higher grade of manhood must bring to his aid the knowledge and skill of the physician and sanitarian, as well as the sympathy and christianity of the devoted missionary.

The population of the Cattaraugus Reservation is about 1,800. The census, which is taken annually by the government, shows that for the past ten years there has been but little variation from this number. The death-rate is very large, as is also the increase by birth.

Of this 1,800, fully 100 die each year from tubercular diseases. Consumption occurs among them from early infancy. There is good reason to believe that one-third of the children born, die of this disease, or of some other form of tuberculosis, before the age of five years. If they pass this age in safety, they are likely to enjoy immunity until they reach puberty, at which time, especially with the females, there is great fatality.

Young men and women, who perhaps a few months before were apparently in good health, come to the dispensary with some indefinite complaint, and upon examination are found with tuberculous lungs. Whole families frequently die of this disease within a few years, and were it not for their wonderful fecundity the race would speedily become extinct from this cause alone. A slight attack of bronchitis, which affection is far more common than with the white race, is sufficient to lay the foundation of phthisis. It is very rare for pneumonia to be followed by recovery, for if the patient survives the acute stage of the disease he almost certainly becomes the victim of acute tuberculosis. The low vitality and consequent inability to resist disease, together with the poverty of resources for proper care, brings the malady to a fatal termination, in most instances within six months from the discovery of its existence.

It may be important to state that the study of tuberculosis upon which the conclusions of this paper are based was not only the result of observation of cases among the Indians living on the Reservation, but was also derived from my opportunities to observe the disease as existing among the inmates of Thomas Asylum for Orphan and Destitute Indian Children. At this institution children are received from two years of age and retained until seventeen. Thus for a long period of years, an opportunity is afforded for the study of the physical condition of each child, and to observe the later history of affections beginning in childhood more accurately than would be possible under most other circumstances.

Tubercular disease among Indian children, according to my observation, exists about as follows: first, in order of frequency stands affected glands, internal and external; next follows tubercular conjunctivitis, with frequent corneal ulcers; then eczema, cold abscess, tuberculosis of the lungs, and, lastly, tuberculosis of the bones and joints.

Many years since I had come to observe among the inmates at the asylum that those who had many glands destroyed by suppuration seemed to escape more serious tubercular trouble. In other words, the enlargement, softening and discharging of the contents of a large number of these glands protected the individual, in the larger number of instances, from consumption. On the other hand, a sufficient number of cases have been seen where absorption has been brought about by the use of iodine and other means locally, to establish the fact that the disappearance of the swollen glands is quite likely to be followed by the discovery of tubercular disease in some other part, generally the lungs. In fact, we became convinced that where many glands were involved and the engorgement extensive it was highly dangerous to promote absorption, and that cases did far better which were allowed to suppurate and after complete softening, opened by free incision, the abscess cavity washed with antiseptic solution, and if slow in healing thoroughly curetted.

An examination of the case book at the Thomas Asylum, made ten years since, showed at that date that there had been received during the preceding seven years 260 children; that out of that number 54 cases of hypertrophy of glands, tubercular in character, occurred. In 35 of these cases the enlargement disappeared by absorption, and was followed in 18 of this number by tuberculosis of the lungs, with 14 deaths. In the remaining 19 cases, profuse and extensive suppuration took place, the glands were entirely destroyed and in 8 cases the abscess cavities were thoroughly curetted. Out of this number there were 4 cases of pulmonary tuberculosis with 2 deaths.

Since the record from which the above tabulation was made, with the exception of two years, a comparatively accurate history of each case of disease occurring at the institution has been made. A review of this history confirms the conclusions herein given. Many children come to the Asylum with enlarged cervical and other glands. Others undergo this condition after becoming inmates. Under the plan of treatment now pursued these children become after a time the most healthful ones undei our care. As before stated, the development of phthisis at puberty is a common occurrence, especially with the girls, but those who have undergone extensive glandular tuberculosis with suppuration, escape. This has been verified in so many instances under my observation, that I feel assured that, when I see a girl with many scars, showing previous tubercular abscess, she will safely pass the critical period of puberty.

The same protection seems to be afforded in tuberculosis of bones and joints. In several instances there have occurred, with long continued suppuration, open sinuses and extensive destruction of bone tissue and joint structure, followed by ankylosis. This last condition, complete as it may become, and lasting for many months, generally in time permits of motion with a resulting useful joint, even with extensive destruction of joint surface.

It is to be noted that these cases do not bear operation well. For instance, it was thought necessary to amputate the hand of a girl fifteen years of age, for extensive necrosis of the carpus. A careful examination of the lungs failed to reveal any disease of those organs, but two weeks subsequent to the operation, she began to cough, her temperature was elevated, and an examination of the chest showed beyond question the existence of tuberculosis, of which she died in a few weeks. We have a girl now in the last stages of consumption, which disease made its appearance after the amputation of the foot.

It would seem that the best results follow, as a rule, in these cases when all the processes necessary for the complete disintegration, separation, and discharge of the diseased tissue are allowed to take place, unimpeded and uninterrupted. Individuals are often seen among these Indians, showing by scars extensive loss of bone, who have evidently never received any treatment, and are now apparently in good health.

In addition to my work at the Asylum, I see at the dispensary on the Reservation, of which I have charge, from twenty to sixty Indians, each week. The chronic affections for which I prescribe there are veiy largely in some manner tubercular. Indeed, I think with very few exceptions the whole population of the Reservation may be divided into two classes: first, those who manifest existing tuberculosis; second, those who have suffered from the affection, as shown by the existence of scars, deformities, etc., and who are now, I believe, immune, and have become the more vigorous men and women of the tribe. It is not uncommon to obtain from parents this history: that out of a large family of children born to them only one or two are now surviving, the others having died of consumption. It is very often to be observed that the surviving children show by the presence of scars that they have suffered from glandular tuberculosis.

My observation of this disease among these people has forced upon me the belief that in tuberculosis, as with several other contagious diseases, recovery from one attack protects against a second invasion.

It would at least seem there is enough evidence that this is true to demand in the study of this great disease more thorough investigation, having in view the important question of prophylaxis.

Literature bearing on the question of how far tuberculosis affecting organs of small importance, may by its invasion and recovery therefrom, afford protection from subsequent tuberculosis of vital organs is, so far as I have been able to ascertain, exceedingly scant. Dr. W. Gilman Thompson, in Pepper's New System, says, after demonstrating the relationship of scrofula and tuberculosis: "It is true that fatal cases of phthisis seldom exhibit scrofulous symptoms or lesions." Dr. B. Rachford says, in the New York *Medical Journal*, "The early diagnosis of glandular tuberculosis in children is important for two reasons: first, it is curable; second, when recovered from, it affords partial protection against pulmonary tuberculosis later in life."

The prevalent belief seems to be otherwise, viz.: that tuberculosis of glands is the precedent of general tuberculosis; that general infection, which may be localised in pulmonary phthisis, is more often than otherwise derived from these so-called scrofulous engorgements. If it be true that tuberculosis is contagious, and few are bold enough to deny this with the cumulative evidence which the past few years has produced; if it can be shown that immunity from further attacks is secured by certain forms of the disease of comparatively mild character, then there is open to the thoughtful physician a wonderful field of labor, looking toward the extinction of the most fearful scourge known to humanity. With the acceptance of these two propositions, there comes before us the interesting possibility of not only curative treatment, but also of the production of artificial immunity, as applied to the disease under consideration.

The study of the protective influence of blood serum containing virulent specific microörganisms, or cultures of the same, or some modification of it, is at present largely engaging the attention of bacteriologists. It has also so far convinced practical men in our profession of its great value, that in rabies, tetanus and diphtheria, it has become an established therapeutic agent.

May we not reasonably hope that the next few years are to see the same plan of treatment successfully applied to tubercular phthisis? When tuberculin had fallen into disuse, many were convinced that at least the embodying principle was correct, and they looked with confidence to the outcome of the further developments of the investigations of Koch, its great discoverer. There can be doubt that the present generation is to witness the fruition of the splendid work of Koch, Pasteur and others in a vastly decreased mortality from many infectious diseases. Among them let us hope is to be included tuberculosis.

